# Medical Opinion and Movement

**Published:** 1910-01-22

**Authors:** 


### Medical Opinion and Movement.

Dr. A. Schanz, of Dresden, draws attention to the Epileptic Convulsions which not infrequently follow orthopædic operations in children. The attacks resemble in all respects infantile epileptic convulsions, and any isolated case would suggest simply a coincidence. It is only a series of such experiences that has led to the conviction of a causal connection between the operation and the attack. According to the investigations of Dr. Von Aberle these convulsions are due to fatty embolism resulting from disturbance of the bone-marrow and passage of the fat into the general circulation. The more severe cases terminate in death immediately after operation with pulmonary symptoms. In milder and more protracted cases cerebral symptoms predominate. For the treatment of this condition Dr. Schanz advocates copious subcutaneous injections of normal saline. As soon as the convulsions appear he injects a half to one litre of normal saline in different parts of the body. He has treated 10 cases in this way, and all have recovered. In one case a hemiplegia developed, but it cleared up in the course of a fortnight. It is supposed that the saline injections act by dilating the capillaries and so allowing a free passage for the fatty globules. The increase in the circulating fluid would also raise the blood-pressure and cause a greater dilution of the fatty emulsion. In very severe cases in which death is threatened either during operation or immediately afterwards the author advises injection directly into the veins.

---

Dr. Graham, of Chicago, recommends the administration of Olive Oil by the mouth to combat the nausea and vomiting following Ether Anæsthesia. Arguing from the fact that substances such as fats, which are soluble in ether, are able to restore to the blood certain properties, such as phagocytosis, more or less affected by the anæsthetic, the idea occurred to Dr. Graham that possibly other effects, such as the nausea and vomiting following ether administration, might also be beneficially influenced in the same way. The results of his treatment on these lines in 30 cases at the Rush Medical College appear to confirm this hypothesis. As soon as the patient commenced to recover consciousness from the anæsthetic olive oil was given in doses of 30 grammes, and in most cases the usual nausea following etherisation did not occur. In other cases in which nausea was already present before the administration of oil the treatment brought about an immediate cessation of the nausea. Only in one case the oil failed to be entirely efficacious. The nausea and vomiting ceased for a while after a dose of oil, but recommenced 10 hours later. Discrimination must, of course, be exercised to ascertain that the nausea and vomiting are of anæsthetic origin, and not due to some other cause, such as peritonitis, microbic intoxication, and the like. Furthermore, only pure olive oil should be used, free from any fatty acids, such as may result from exposure to the air or from sterilisation.

Dr. Siebert, of St. Francis Hospital, New York, advocates Subcutaneous Injections of Camphorated Oil in large doses in the treatment of Pneumonia. Such injections are not infrequently used, especially in Germany, to combat cardiac failure in the later stage of the disease; but Dr. Siebert claims that when given in sufficient doses the drug acts directly upon the morbid causal agent and considerably modifies the course of the disease. The treatment should be commenced as early as possible, and the camphorated oil, of 20 per cent. strength, should be injected in doses of 12 c.c. or more every 12 hours until the temperature, pulse, and respiration are reduced to normal, and then the interval may be lengthened to 24 hours, but the injections should be continued till the lung is completely clear of the disease. Dr. Siebert gives a detailed account of several cases in which the effects of the injections on the temperature, pulse, and respiration are most striking. The author has treated altogether 21 cases of pneumonia in this way, and all have recovered. Although this may be considered in itself insufficient proof of the efficacy of the treatment, the direct effect of the injections upon the disease is seen in the fact that, according to the author, under this treatment the natural crisis of pneumonia is entirely absent. In its place there is a steady improvement of all the symptoms, which is increased after each injection, so that in the end the duration of the disease is considerably diminished. Experiments in the laboratory show that the development of the pneumococcus is arrested in cultures by the addition of camphor in the strength of 1 in 10,000. Further, a certain dose of pneumococcus in emulsion injected into a rabbit's ear results in the death of the animal in 36 hours. If the same dose of pneumococcus be followed by a subcutaneous injection of 1 c.c. of 20 per cent. camphorated oil an hour afterwards and the injections of oil are repeated every 12 hours the animals recover in two days. It appears, therefore, that the camphor circulating in the blood is able to destroy the pneumococcus, or at least arrest its morbid effects.

---

Dr. Mink, a Dutch physician, has conceived a new method of Diagnosing Pus in the Antrum of Highmore by means of Auscultation. He points out that diaphanoscopy is subject to errors, and radiography is not always available, whereas auscultation affords a ready and easy method of diagnosis. The auscultation can be carried out by means of an indiarubber tube with a suitable earpiece attached at one end and an otoscope at the other end. The otoscope is applied to the maxillary bone at the canine fossa and the patient is directed to breathe quietly. If the sinus is clear, a typical amphoric murmur is heard, which is accentuated by increased respiration. This murmur does not originate in the nose, as might be suggested, as it is still present, although diminished, when the maxillary orifice is obstructed by means of a pledget of cotton wool. When the antrum is full of fluid nothing is heard on auscultation. In a patient with a communication into the antrum through an alveolus this can be confirmed by injecting fluid into the antrum and then auscultating. Negative auscultation does not necessarily signify fluid in the antrum. It may be occupied by a tumour, or again nothing is heard in the case of obstruction of the nasal fossa by a polypus or by swelling of the mucous membrane. It should be ascertained, therefore, that the nasal fossa is free, and the author recommends the application of cocaine and adrenalin solution first to ensure a clear passage.

---

Epididymo-Vasostomy is the name given by Dr. E. Martin, of Philadelphia, to an operation for the cure of sterility caused by obliterating double epididymitis. Of this he reports in the *Therapeutic Gazette* fifteen cases, some of them most strikingly successful. He finds that the testis, contrary to the usual rule with glands, does not suffer any impairment of its secreting power when its duct is blocked up, even for many years. Although the spermatozoa in their passage through the normal epididymis undergo changes which can be demonstrated microscopically, this phase does not seem to be essential to their proper development, as they are quite capable of fertilising an ovum without passing through the epididymis. Recurrent bilateral epididymitis is more likely to produce real azoospermia than obliterative obstruction of the duct: it is only the latter that can be remedied by operation. Also the obstruction may not be in the epididymis at all, but in the vas or ejaculatory ducts: in such cases anastomosis between the vas and the epididymis or the testicle is not likely to succeed. The point can be tested by exposing the vas under local anæsthesia, and injecting carmine into it. In six cases out of fifteen spermatozoa have reappeared in the semen of those operated on, and in three of these a sterile marriage has been rendered fertile. The technique of the operation is said to be quite simple: local anæsthesia can be made to suffice, but is not recommended. With a proper supporting arrangement the patient can follow his occupation as soon as he recovers from the anæsthetic.

---

The Ætiology of Heberden's Nodes is discussed by Dr. J. B. Burt in the *Medical Chronicle*. He says that these curious bony prominences, which are formed of true bone and covered by an extension of the articular cartilage, are always preceded by some condition which leads to the weakening of the joint ligaments. The more the joint is used, the liability to develop nodes is greater, but they occur in a variety of conditions which are in no way connected with one another. The most important of these are gout, chronic rheumatoid arthritis, and old age. The author has observed that the outgrowths are always situated on the posterior margin of the joints affected, and accounts for this by the extra and intermittent pressure exerted on the bone posteriorly by the usual movements of opposition of the fingers when the ligaments are weakened by disease or senile degeneration. The hypertrophy thus caused of the most prominent portions of the margins which sustain this intermittent strain constitutes Heberden's nodes. Considered from a mechanical point of view they serve a useful purpose, for the pressure is distributed over an increased surface. In a word, Dr. Burt regards these prominences as a compensatory change, and not a sign of one specific disease.

---

Some Obscure Problems in connection with Hepatic Cirrhosis are dealt with by Dr. A. T. Wilkinson. He points out a fact not always remembered, that a very large percentage of the cases occur in patients who are not drunkards, though they may be, and usually are, free drinkers. Many a man develops cirrhosis after a few years' steady indulgence in a daily quantity of alcohol which he himself regards as quite moderate: he may never even have been drunk in his life. On the other hand, many men live to 70 or 80, although they seldom go to bed sober, and even then do not die of cirrhosis. Against the alcoholic-gastric-catarrh theory of the disease he sets the fact that many subjects of cirrhosis never have any dyspeptic symptoms, and their gastric mucous membranes are found quite normal post-mortem. Cirrhosis, in fact, he has found for the most part—though, naturally, not invariably—among those who have no digestive troubles. Why is it that, in spite of the free collateral circulation established between the portal and general systems, sugar is practically never found in appreciable quantity in the urine? Why, without apparent heart failure or pressure on the vena cava, do patients develop simultaneously ascites and œdema of the legs? Why does the spleen vary so in size? And, lastly, why do sensible men still exceed? This question is as difficult to answer as the others, and Dr. Wilkinson does well to point out that this class of men will frequently listen to a doctor when they will pay no attention to a clergyman, and that a corresponding responsibility rests upon us which it should be our privilege to assume.

---

Some very Pertinent Criticisms of Urinary Surgery as at present practised are contributed by Mr. J. G. Pardoe to the *West London Medical Journal*. Suprapubic prostatectomy, for instance, is an operation the enormous value of which in properly selected cases is so well recognised that it is now also practised, in his belief, without real justification. Especially is this the case in regard to carcinoma of the prostate; in not a single case of this condition has he ever known benefit to result from enucleation, and in almost all cases actual harm follows it. The significance of this statement is obvious when it is remembered that one of the best authorities on enlarged prostate encounters malignant disease in about 10 per cent. of his enucleations. Carcinoma of the prostate is a very chronic and slow disease if left alone; not so after injudicious interference. Unfortunately it is so insidious in onset that a positive diagnosis is too often only to be made in advanced and hopeless cases. It follows that carcinoma is frequently met with in prostates supposed to be adenomatous, and it may be only upon microscopic examination that the nature of the growth is recognisable. In any doubtful case where the slightest suspicion of malignancy is entertained an exploratory incision in the perineum is recommended for the removal of a small piece of the gland; should the report show carcinoma, a really radical removal of the gland with the seminal vesicles and the lower part of the bladder is the only justifiable operation, and if this cannot be carried out no surgical measures beyond catheterisation and, if necessary, suprapubic cystostomy are admissible.

---

With the Argyll-Robertson pupil, which reacts to accommodation but not to light, everyone is familiar. The converse of this, a Pupil which Reacts to Light but not to Accommodation, is much rarer. Such accommodative iridoplegia has been described by three observers during 1909 in cases of diphtheritic paralysis, a condition in which apparently it has not been observed before. Two of the cases were seen in this country (by Dr. H. H. Tooth and Mr. S. Stephenson respectively) and the third at Munich by a local ophthalmologist. The condition is unmentioned even in the most exhaustive textbooks. The details of Mr. Stephenson's case are given in the *Ophthalmoscope*. The patient is a boy of nine years old who had suffered from an "ulcerated throat" which was not treated with antitoxin because of negative bacteriological findings. Five weeks later he was found to be pronouncing nasally and to have occasional regurgitation of fluids. The knee-jerks were almost, but not quite, lost; but there was no other paretic lesion except that in the iris. Ocular movements, including convergence, were good, and the pupils reacted well and promptly to light. Accommodation was practically lost, as a + 7 D sphere was required for reading No. 1 Jaeger type at 20 cm. The fundi were normal. The treatment adopted comprised rest in bed and abstention from reading, with strychnine and phosphoric acid as medicines. In six weeks every sign of paresis disappeared, except that the knee-jerks remained rather sluggish. Accommodation and the accommodation reflex both returned to normal. Mr. Stephenson suggests that this symptom might be more often found if systematically searched for.

---

In the *Edinburgh Medical Journal*, Dr. G. A. Gordon emphasises the value of Accurate Blood-pressure Estimations. His article considers the fluctuations which may be exhibited in many diseases: perhaps his observations on cardiac cases are more interesting than on some of the other lesions dealt with. In aortic incompetence the systolic pressure is very high, but the diastolic pressure is low; the difference between them, which he terms the Pulse Pressure, is therefore much greater than in health. In aortic stenosis, on the contrary, the systolic pressure is low, the diastolic relatively high; consequently, the pulse pressure is much decreased. In mitral lesions the exact state of the valvular opening has less bearing on the pulse than have such symptoms as dyspnœa, cyanosis, œdema, etc. These conditions raise both systolic and diastolic pressure by stimulating the vasoconstrictor centre in the medulla; this is brought about by the excess of carbonic acid gas contained in the blood during these states. When, therefore, in mitral stenosis or incompetence there is cyanosis, both systolic and diastolic pressures are high and the pulse pressure is low; when there is no cyanosis both factors are variable, and therefore the resultant is also. In myocarditis systolic and diastolic pressure are both high, but the former predominates and the pulse pressure is increased; should the heart be dilated, however, this no longer holds, for then the pulse waves are low and the pulse pressure is also decreased. In "heart-block" the systolic pressure is generally enormously increased, while the diastolic is raised also, but not to nearly such an extent. The pulse pressure is greatly increased, and this has been supposed to be due to the long intervals elapsing between the contractions of the ventricles. These observations have, the author points out, a practical application in diagnosis; thus an obscure presystolic murmur which may be due to mitral stenosis, or may be a Flint's murmur of aortic reflux, can be assigned with some confidence to one cause or the other by taking the pulse pressure.

---

In Pneumonia also the author attaches great importance to systematic observations of the blood-pressure. Gibson was the first to remark that, when the arterial pressure, expressed in millimetres of mercury, does not fall below the pulse rate, expressed in beats per minute, the prognosis is good, whereas when it does the prognosis is bad. Dr. Gordon specially tested this point in fifteen cases, and the results were strikingly confirmatory of the proposition. Whenever the pressure thus reckoned exceeded the pulse rate, a favourable result ensued. In one case, where the pressure fell to below the pulse rate, yet the patient recovered; but this was only achieved by persistent and bold stimulation, and the day before the crisis the blood-pressure reacted to the measures adopted by rising above the pulse rate for the first time. The author advocates that in every case of pneumonia a chart should be made to show the relative blood-pressures and pulse rates. Thus the first signs of failing heart can be detected long before the pulse or the cardiac sounds give any indication of it. Stimulation can then be begun before it is too late, and heart tonics should be administered just to that degree necessary to keep the blood-pressure higher than the pulse rate. Unless this can be done, recovery will seldom occur. If further research confirms these observations, a case will certainly have been made out for the use of the sphygmomanometer in pneumonia, and also for the use of copious stimulation, to which a few physicians still object.

